# Further Mulinane and Azorellane Diterpenoids Isolated from *Mulinum crassifolium* and *Azorella compacta*

**DOI:** 10.3390/molecules19043898

**Published:** 2014-03-28

**Authors:** Jorge Bórquez, Alejandro Ardiles, Luis Alberto Loyola, Luis Manuel Peña-Rodriguez, Gloria María Molina-Salinas, Javier Vallejos, Isidro G. Collado, Mario J. Simirgiotis

**Affiliations:** 1 Laboratorio de Productos Naturales, Departamento de Química, Universidad de Antofagasta, Av. Coloso S-N, Antofagasta 1240000, Chile; E-Mails: jorge.borquez@uantof.cl (J.B.); euro_ale@hotmail.com (A.A.); alberto.loyola@uantof.cl (L.A.L.); 2 Grupo de Química Orgánica, Unidad de Biotecnología, Centro de Investigación Científica de Yucatán, Calle 43 No 130, Colonia Chuburná, Mérida, Yucatán 97200, Mexico; E-Mail: luispenarodriguez@yahoo.com; 3 División de Biología Celular y Molecular, Centro de Investigación Biomédica del Noreste, Instituto Mexicano del Seguro Social, San Luis Potosí y Dos de Abril, Colonia Independencia, Monterrey, Nuevo León 64720, Mexico; E-Mail: gmolina70@gmail.com; 4 Unidad de Alta Especialidad Médica Yucatán Lic. Ignacio García Téllez Calle 41 No 439 x 32 y 34, Col. Industrial, CP 97150, Mérida, Yucatan 97200, Mexico; 5 Departamento de Química, Universidad Católica del Norte, Av. Angamos 610, Antofagasta 1240000, Chile; E-Mail: jvallejos01@ucn.cl; 6 Departamento de Química Orgánica, Facultad de Ciencias, Universidad de Cádiz, Apartado Postal 40, Puerto Real 11510, Spain; E-Mail: isidro.gonzalez@uca.es

**Keywords:** mulinanes, azorellanes, new diterpenoids, Chilean native plants, semisynthesis

## Abstract

The new mulinane diterpenoids **1** and **2** were isolated from the EtOAc extract of *Mulinum crassifolium*, while the rearranged mulinane **5**, which was isolated for the first time from a natural source, was isolated from *Azorella compacta*. Compounds **1**–**2** were prepared by semi-synthesis thorough acetylation of the diterpene 17-acetoxymulinic acid (**3**). A mechanism of reaction was proposed, while the structures of the new compounds were elucidated on the basis of comprehensive spectroscopic analysis and computational methods.

## 1. Introduction

**Figure 1:**
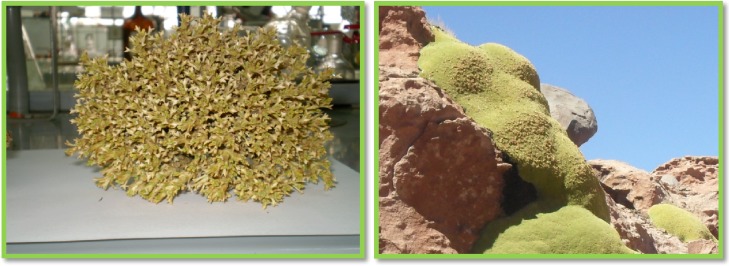
Pictures of *Mulinum crassifolium* (left) and *Azorella compacta* (right), from Northern Chile. (Taken by Jorge Bórquez and Mario J. Simirgiotis, on March 2011).

**Figure 2:**
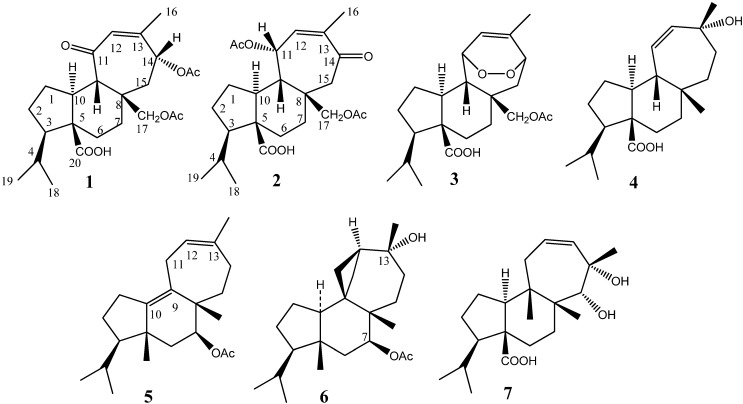
Structures of the diterpenoids isolated from *Mulinum crassifolium* and *Azorella compacta.*

*Mulinum crassifolium* is a 15 cm small cushion shrub, which grows in the north of Chile at altitudes above 4,000 m. This plant (Figure 1 left), commonly known as “chuquican” or “espinilla” is used in folk medicine, principally against diabetes and bronchial and intestinal disorders [1]. *Mulinum* *crassifolium* is well recognized as an important source of diterpenes bearing the mulinane skeleton, while *Azorella compacta*, another cushion shrub known as “llareta” (Figure 1 right) is a source of diterpenoids with both the mulinane and azorellane skeletons [2,3,4]. There is a phylogenetic relationship between these two shrubs, both belonging to the group *Azorella* (Apiaceae). This group includes six genera (*Azorella*, *Laretia*, *Schizeilema*, *Mulinum*, *Huanaca*, and *Stilbocarpa*) with Austral-Antarctic affinities and a geographic distribution that extends from Oceania to South America. The “*Mulinum* clade” is further divided into six subclades, which collectively include 23 of 26 species of *Azorella*, the monotypic *Laretia* and all species of *Mulinum* [5]*.* The rare diterpenoids isolated from these genera have displayed a wide variety of interesting biological activities, including trypanosomicidal [6], trichomonicidal [7], toxoplasmocidal [8], antiplasmodial [9], antibacterial [10], spermicidal [11], antihyperglycemic [12], antitubercular [13], antiinflamatory and analgesic activities [14,15]. In our continuing efforts in the search for the structurally diverse and biologically important metabolites from the genus *Azorella* [4,7,16] and *Mulinum* [4], two new mulinane diterpenoids, named mulinones a-b (compounds **1** and **2**, Figure 2) were isolated from *M. crassifolium* together with 17-acetoxymulinic acid (**3**) and mulinolic acid (**4**). The rearranged mulinane **5**, which is a new natural product but was previously reported as a reaction product [15], together with the known compounds azorellanol (**6**), and 13,14-dihydroxymulin-11-en-20-oic acid (**7**), were isolated from *A. compacta*. Compounds **1**, **2** and **5** [14,15] were synthesized from their precursor natural products**.**

This paper deals with the isolation and structural elucidation of the new compounds. Furthermore the semi-synthesis of compounds **1**, **2** from the parent compound **3** (Figure 2) and the proposed mechanism of reaction were addressed. 

## 2. Results and Discussion

### 2.1. Isolation and Structural Elucidation of the New Diterpenoids

From the ethyl acetate extracts of the aerial parts of the Chilean cushion shrubs *Mulinum crassifolium* and *Azorella compacta* two new diterpenoids (compounds **1** and **2**, Figure 2) were isolated together with five known compounds (compounds **3**–**7**, Figure 2, for details please see the experimental part). These new compounds along with the new natural product **5** were semi-synthesized; compounds **1**–**2** from 17-acetoxymulinic acid **3**, while compound **5,** which is isolated for the first time from a plant, was previously synthesized from the azorellane diterpenoid **6** [15]. This fact confirmed their precursor natural products. The compounds isolated were proved not to be artifacts of the extraction procedure with ethyl acetate by extracting a portion of the plant material in the presence of calcium carbonate (0.1%). The same natural products were detected and isolated using this procedure. The structural elucidation of the new compounds and proposed mechanism of reaction is explained below. 

HREIMS of **1** showed an ion consistent with a molecular formula of C_24_H_34_O_7_ (required: *m/z* 434.2304, found 434.2284). The total of 24 carbons suggested the presence of a diacetylated diterpene. The ^1^H-NMR and ^13^C-NMR spectral data were easily assigned by comparison with NMR data of related compounds and in particular with the parent compound 17-acetoxymulinic acid [9,17,18] (Table 1 and Table 2, Figures S1–S8, Supplementary Material). The chemical shift values of the cyclopentane and cyclohexane rings were practically identical, confirming the configuration of the stereogenic centers and substitution pattern. The remaining signals indicated the presence of a trisubstituted double bond in the seven membered ring (δ_C-12_ 127.1, d, and δ_C-13_ 150.5, s), together with the corresponding signal of one vinylic proton (δ_H-12_ 5.84 brs), a secondary acetoxyl group (δ_H-14_ 5.75, dd, *J* = 11.3, 3.8; δ_C-14_ 71.5, d) and a ketone (δ_C-11_ 202.6, s). HMBC cross-peaks of the proton at δ_H_ 2.63 (H-9) with C-11 (202.6) and C-12 (127.1) suggested the presence of an α,β-unsaturated ketone. The methyl group CH_3_-16 showed HMBC correlations to three carbons (C-12, C-13 and C-14) which confirmed the position of the double bond. The methyl group at δ_H_ 2.11 (3H, s, H-22) showed cross-peaks with C-21 and C-14, while H-14 (δ_H_ 5.75) showed cross peaks with C-12, C-13 and C-15, which further confirmed the position of the additional acetyl group on C-14. The main results from phase sensitive NOESY spectra (Table 1, Figure S7) suggested that **1** had the stereochemistry shown in Figure 2. Proton H-14 showed dipolar correlations with H-17 and H-9 protons, both with β configuration; this fact confirmed the α-orientation of the acetyl group on C-14. All the spectroscopic evidence stated above proved that **1** has the relative stereochemistry as proposed. 

**Table 1:** ^1^H-NMR data, HMBC and NOE correlations for compounds **1** and **2** in CDCl_3_ (*J* in Hz in parentheses).

		1			2	
Proton	δ_H_ mult. (*J* in Hz)	HMBC (H→C)	NOE	δ_H_ mult. (*J* in Hz)	HMBC (H→C)	NOE
1α	1.24 m	C-5, 9		1.83 m	-	-
1β	1.84 dd (11.4, 4.7)	-		1.37 m	-	-
2α	1.97 m	-		1.79 m	-	-
2β	1.58 m	-		1.48 m	-	-
3α	1.48 m	-		1.35 m	C-2, 4, 5, 18, 19	H-10α
4	1.54 m	-		1.45 m	-	-
6α	1.43 m	-		1.22 m	-	H-10α
6β	1.52 m	-		2.45 dt (7.2, 4.0)	C-5, 8	H-7β, Me-19
7α	2.56 dt (13.3, 3.2)	C-15, 9, 17		1.80 dd (13.6, 4.5)	-	-
7β	1.59 m	-		1.71 dt (14.3, 4.3)	C-8, 9, 15	H-6β, H-7β,
9β	2.63 m	C-7, 15, 10, 11		2.23 m	C-8, 10, 11, 12, 15	H-11β, H-17β
10α	2.22 m	C-20, 9, 5, 8, 6	H-1α, H-6α, H-3α	1.51 m	C-1, 5, 9	H-6α, H-3α
11β	-	-		5.94 br d (2.4)	C-8, 9, 10, 12, 21	H-9β, H-17β
12	5.84 br s	C-9, 13, 14		6.20 br s	C-9, 13, 14, 16	H-11, H-16
14	5.75 dd (11.3, 3.8)	C-12, 13, 15	H-17β, H-15β, H-9β	-	-	-
15α	261 dd (14.1, 11.3)	C-14, 7, 17, 13		2.62 d (12.7)	C-14, 7, 17, 13, 8	-
15β	1.68 dd (14.1, 3.8)	-		2.50 d (12.7)	-	-
16	1.85 br s	C-13, 12, 14		1.87 br s	C-13, 12, 14	-
17α	3.85 d (11.3)	C-9, 7, 15		3.94 d (11.1)	C-8, 9, 15	-
17β	4.17 d (11.3)	-	H-14β, H-15β	4.00 d (11.1)	-	H-9β, H-11β
18	0.85 d (5.5)	C-4, 3		1.99 d (6.3)	C-4, 3	-
19	1.02 d (5.5)	C-4, 3		0.82 d (6.3)	C-4, 3	-
22	2.11 s	C-21, 14		2.09 s	-	-
24	1.99 s	C-23, 17		2.04 s	C-23, 17	-

**Table 2:** ^13^C-NMR data (100.25 MHz) for the new compounds **1** and **2**.

C#	1	2	C#	1	2
1	24.2 t	25.7 t	13	150.5 s	138.1 s
2	28.4 t	28.5 t	14	71.5 d	201.7 s
3	57.5 d	57.3 d	15	36.2 t	52.5 t
4	31.6 d	31.6 d	16	28.4 q	18.5 q
5	57.0 s	56.1 s	17	71.6 t	71.0 t
6	31.6 t	32.7 t	18	22.7 q	22.6 q
7	36.0 t	34.3 t	19	22.3 q	22.3 q
8	46.8 s	37.9 s	20	178.4 s	178.1 s
9	57.4 d	45.9 d	21	170.5 s	170.7 s
10	46.9 d	47.3 d	22	20.9 q	21.2 q
11	202.6 s	72.2 d	23	169.5 s	169.9 s
12	127.1 d	139.2 d	24	20.5 q	20.8 q

The diterpenoid **2** had the same molecular formula (C_24_H_34_O_7_, (required: *m/z* 434.2304, found 434.2348), as **1**, and showed a similar IR spectrum. The ^13^C-NMR spectrum exhibited 24 peaks (Table 2) while the DEPT-135 showed evidence for the same numbers of methyl, methylene, methine and quaternary (including four carbonyls) carbons, as compound **1**. However, in the ^1^H-NMR spectra the simplification in the coupling pattern of the methylene protons (δ_H15α_ 2.62, d, *J =* 12.7 and δ_H15β_ 2.50, d, *J* = 12.7) of the cycloheptane ring as well as the strong deshielding of the beta oriented one which appeared as a broad doublet (δ_H11_ 5.94, brd, *J =* 2.4) suggested that the location of the keto carbonyl group was at C-14. Moreover, in the ^13^C-NMR spectrum of compound **2**, C-14 showed a big upfield shift δC_14_ = 201.7), whereas C-11 showed a downfield shift (δC_11_ = 72.2), this suggested that the keto group is placed at C-14 while the acyl group is at C-11 (See Figures S9–S16). The structure of **2** was confirmed by careful analysis of the HMBC spectra (Table 1, Figure S16). Strong correlations were observed between the signal at δ_H_ 1.87 (3H, s) assigned to CH_3_-16, and signals at δ_C_ 139.2 (d, olefin C-12), δ_C_ 138.1 (s, olefin C-13) and δ_C_ 201.7 (s, carbonyl C-14). The signals at δ_H_ 2.62 and 2.50 assigned to H-15α and H-15β correlated with the signal at δ_C_ 201.7 (s, carbonyl C-14), δ_C_ 138.1 (s, olefinic C-13) and 71.0 (t, CH_2_OAc, C-17). These facts confirmed the location of the keto group at C-14 and the assignment of a double bond between C-12 and C-13. The location of the acetyl groups at C-17 and C-11 was confirmed by the correlation of the protons CH_2_OAc (C-17, δ_H_ 3.94 and 4.00, both, d, J = 11.1 Hz) with C-8, C-9 and C-15 and the proton CHOAc (C-11, δ_H_ 5.94, brd, J = 2.4 Hz) with C-8, C-9, C-10 and C-12. Hence, the relative stereochemistry of **2** corresponds to mulin-12-en-11α,17-diacetoxy-14-one-20-oic acid (Figure 2). 

### 2.2. Semisynthetic Procedures and Proposed Mechanisms of Reaction

Compounds **1**, **2** and **5** were prepared from their precursors **3** and **6**. The reactions are explained below. 

#### 2.2.1. Semi-Synthesis of Compounds **1** and **2**

**Figure 3:**
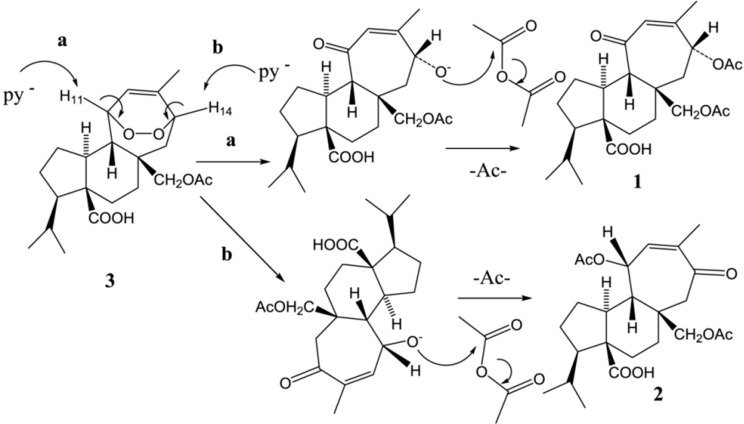
Proposed rearrangement of **3** with Ac_2_O in pyridine at 25 °C.

Treatment of **3**, with pyridine-Ac_2_O, at 25 °C under inert atmosphere yielded compounds **1** and **2**. The proposed mechanism of the reaction is shown in Figure 3. The two reaction pathways **a**–**b** shown for compound **3** can be explained by a mechanism in which the base pyridine abstracts a proton from either the **a** or **b** sides (H_11_ or H_14, _respectively, Figure 3) of the mulinane skeleton leading to the opening of the endoperoxide ring with simultaneous formation of an α-β-unsaturated carbonyl followed, by acylation of the remaining oxygen anion with Ac_2_O leading to the mulinane compounds **1** and **2** (79.2% and 13% yield, respectively). 

#### 2.2.2. Computational Analyses and Mechanism of Reaction

To explain the difference in the reaction yields for compounds **1** and **2**, two possible transition states TS_a_ y TS_b_ were designed according to the mechanism proposed (Figure 3) to investigate the activation energy for the reactions pathways **a** and **b**.

**Figure 4:**
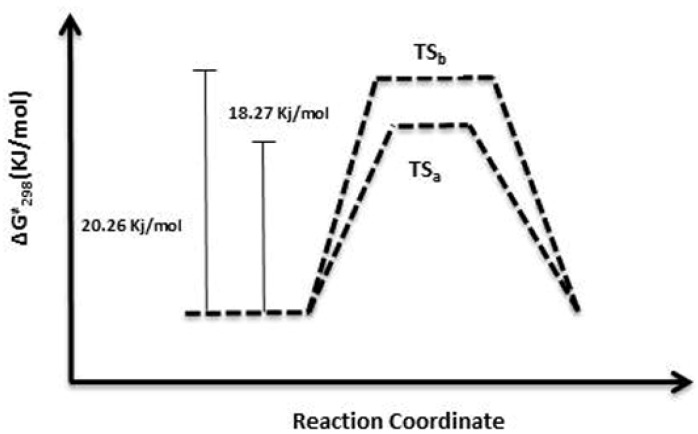
Reaction coordinate (reaction steps **a**, and **b**) calculated (Gaussian 09) [19] for the reaction between mulinane **3** and pyridine leading to compounds **1** and **2**.

**Figure 5:**
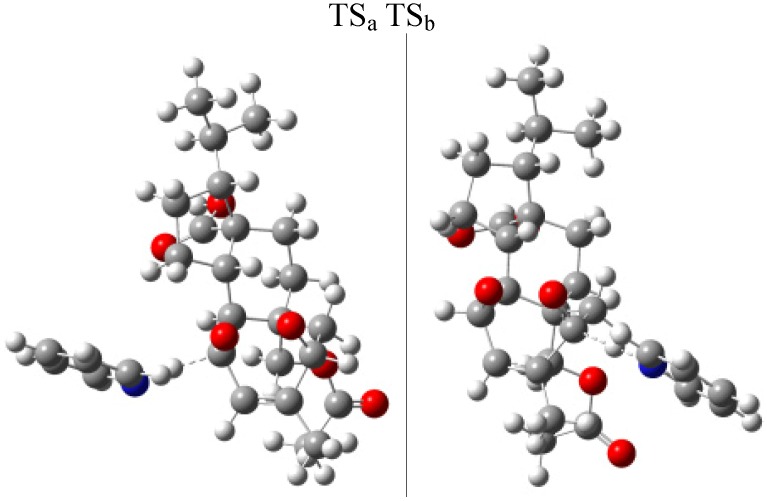
Transition states (TS_a_ and TS_b_, for the reaction steps **a**, and **b**) calculated (Gaussian 09) [19] for the reaction between mulinane **3** and pyridine leading to compounds **1** and **2**.

The geometries of the reactant (compound **3**), and transition states (TS_a_ and TS_b_) for the two reaction pathways (Figure 3), were optimized using the Gaussian software version 09 software package at the B3LYP/3-21G* level [19]. The structures and imaginary frequencies of transition states were confirmed by the vibration analysis and the intrinsic reaction coordinate (IRC) method at the same level. In pathway **a**, the activation energy barrier TS_a_ is 18.27 kJ/mol, while in pathway **b** the activation energy barrier TS_b_ is 20.26 kJ/mol (Figure 4). This difference can explain why the base pyridine takes a proton from both **a** or **b** sides in parallel to produce compounds **1** and **2**. However, the lower energy of TS_a _would reflect a hindered approach of pyridine to the proton H_14_ in side **b** and could explain the higher yield for compound **1** (pathway **a**). Furthermore the higher acidity of leaving proton H_11_ of side **a** compared to the acidity of leaving proton H_14_ in side **b** (the calculated positive charge of H_11_ using the Gaussian 09 software is three times higher than H_14_) proved that the reaction step **a** was favored. This fact was further supported by the calculated transition states for the reaction of mulinane **3** with pyridine. Figure 5 shows the transition states for the subtraction of proton H-_11_ (TSa) or proton H-14 (TS_b_) by pyridine and breakage of the endoperoxide as postulated.

#### 2.2.3. Semi-Synthesis of Compound **3**

The new natural product **5** was also obtained by semi-synthesis from azorellane **6** by stirring this compound in CHCl_3_ at 25 °C for 48 h, until compound **6** was undetectable by TLC, undergoing the mulinane-azorellane rearrangement reported previously [10,14,15]. This reaction proves that the new natural product **5** can be produced from **6** in the plant. 

## 3. Experimental

### 3.1. General Experimental Procedures

Infrared (IR) spectra were recorded on a 783 FTS 165 FT-IR spectrometer (Perkin Elmer, Waltham, MA, USA).^1^H and ^13^C-NMR spectra were recorded on an Avance DRX 500 spectrometer (Bruker Biospin Gmbh, Rheinstetten, Germany) using TMS as internal standard. EIMS and HREIMS spectra were obtained using a MAT 95 XL Mass Spectrometer (Thermo Finnigan, San Jose, CA, USA). Thin-layer chromatography was performed using aluminum-coated silica gel plates (Kieselgel F_254_, Merck, Darmstadt, Germany) with hexane/EtOAc (9:1) as solvent system. The chromatograms were visualized under UV light (254 nm) and then sprayed with 1% vanillin in EtOH (*w/v*) and heated (60 °C) to see the compound spots. Medium pressure column chromatography was performed using Kieselgel 60 H (Merck), 55 μm particle size, FMI QG 150 medium pressure lab pumps (Syosset, NY, USA) and Ace Glass Inc. medium pressure columns (Vineland, NJ, USA).

### 3.2. Plant Material

*Mulinum crassifolium* Phil. and *Azorella compacta* Phil. were collected in *El Tatio*, Antofagasta, Chile in March 2011. Voucher herbarium specimens are deposited at the Laboratory of Natural Products, University of Antofagasta with the numbers Ac-031511 and Mc-031511, respectively.

### 3.3. Extraction and Isolation

Dried and powdered aerial parts of *Mulinum crassifolium* (630 g) were defatted with *n*-hexane (3 times, 1 L each time, 1 day/extraction) and the remaining plant material was extracted with ethyl acetate (3 times, 1 L each time, 1 day/extraction) at room temperature for one day each. After filtration, the solvent was evaporated *in vacuo* yielding a gum (17 g). The concentrated extract was fractionated on a medium pressure silica gel column (5 cm × 20 cm) with hexane-ethyl acetate mixtures of increasing polarity as elution solvents to give six fractions (1–6). Fraction 3 (355 mg) was further separated and purified by medium pressure silica gel chromatography (2 cm × 20 cm) to give 72.2 mg of 17-acetoxymulinic acid (**3,** Figure S17). Fraction 4 (266 mg) was rechromatographed as above to give compounds **1** (95.6 mg) and **2** (15.86 mg). Fraction 5 (327 mg) was re-chromatographed as above to give 42.6 mg of mulinolic acid (**4**) [17]. Dried aerial parts of *Azorella compacta* (745 g) were defatted with *n*-hexane (3 times, 1 L each time, 1 day/extraction) and the remaining plant material was extracted with ethyl acetate (3 times, 1 L each time, 1 day/extraction) at room temperature for one day each and concentrated as stated above to yield 7.2 g of a brown gum. The mulinane diterpenoid **5** (25.6 mg), together with the known compounds azorellanol (**6**, 293.4 mg) and 13,14-dihydroxy-mulin-11-en-20-oic acid (**7**, 74.2 mg) were isolated from this extract by medium pressure column chromatography (Kieselgel 60 H, 5 cm × 20 cm) using the solvent system mentioned above. The structures of the new and known compounds were elucidated by MS and 1D-2D NMR and comparison with literature data [4,9,17,18].

### 3.4. Semisynthesis of 14α,17-Diacetoxymulin-12-ene-11-one-20-oic acid (1) and 11α,17-Diacetoxy-mulin-12-en-14-one-20-oic Acid (**2**)

A portion (50 mg, 0.128 mmol) of **3** was dissolved in a mixture of Ac_2_O/Py (1:1, 2 mL); the mixture was stirred at room temperature for 24 h under N_2_. Afterwards Et_2_O (10 mL) was added, and the Et_2_O fraction was washed with copper sulphate solution (4 × 10 mL) and dried over anh. Na_2_SO_4_. Solvent was evaporated and the remaining reaction products purified by silica gel column chromatography to afford **1** (39.6 mg, 79.2%) and **2** (6.5 mg, 13.0%). 

*14α,17-Diacetoxymulin-12-ene-11-one-20-oic acid* (mulinone **a**, **1**). White crystals, mp 177–178 °C; 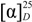 = + 35°, (*c* = 0.200, CHCl_3_); IR (liquid film) _νmax_ (cm^−1^): 3000–3100 (br); 1650, 1700, 1740, 1220; EIMS *m/z* (rel. int.): 434 [M^+^] (10), 392 [M^+^–COCH_3_] (100), 374 [M^+^–AcOH] (7), 314 [M^+^–2 AcOH] (45), 286 (44), 243 (35), 91 (34); HREIMS *m/z*: found 434.2284 (calcd for C_24_H_34_O_7_, 434.2304); ^1^H-NMR (500.13 MHz) and ^13^C-NMR (125.76 MHz), data (CDCl_3_) see Table 1 and Supplementary Material.

*11α,17-Diacetoxymulin-12-ene-14-one-20-oic acid* (mulinone **b**, **2**). White crystals, mp 185–186 °C; 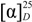 = −28.11°, (*c* = 0.280, CHCl_3_); IR (liquid film) _νmax_ (cm^−1^): 3000 (br), 1700, 1710, 1730, 1230; EIMS *m/z* (rel. int.): 434 [M^+^] (10), 392 (100), 350 (10), 331 (5), 286 (44), 169 (17), 105 (18), 91 (25), 55 (38); HREIMS *m/z*: found 434.2349 (calcd for C_24_H_34_O_7_, 434.2304); ^1^H-NMR (500.13 MHz) and ^13^C-NMR (125.76 MHz), data (CDCl_3_) see Table 1 and Supplementary Material. 

## 4. Conclusions

Based on full spectroscopic evidence, **1** and **2** are new compounds which were named mulinones a-b. Compounds **1** and **2** were synthesized thorough acetylation from **3** in one step. The mechanism of reaction proposed for the semisynthesis of these diterpenoids was explained by computational analyses. This is the first report of the occurrence of diterpenoid **5** in a plant source. These new compounds were semi-synthesized from their precursor natural products—compounds **1**–**2** from 17-acetoxymulinic acid **3** and compound **5** from the azorellane diterpenoid **6**—proving the relationship between them and their parent natural products.

## Supplementary Materials

Supplementary materials can be accessed at: http://www.mdpi.com/1420-3049/19/4/3898/s1.

## References

[1] Munizaga C., Gunkel H. Notas etnobotánicas del pueblo Atacameño de Socaire. http://www.memoriachilena.cl/archivos2/pdfs/MC0038217.pdf.

[2] Chiaramello A.I., Ardanaz C.E., Garcia E.E., Rossomando P.C. (2003). Mulinane-type diterpenoids from mulinum spinosum. Phytochemistry.

[3] Colloca C.B., Pappano D.B., Bustos D.A., Sosa V.E., Baggio R.F., Garland M.T., Gil R.R. (2004). Azorellane diterpenes from Azorella cryptantha. Phytochemistry.

[4] Loyola L.A., Borquez J., Morales G., Martin A.S. (1997). Diterpenoids from Azorella compacta. Phytochemistry.

[5] Nicolas A.N., Plunkett G.M. (2012). Untangling generic limits in *Azorella*, *Laretia*, and *Mulinum* (Apiaceae: Azorelloideae): Insights from phylogenetics and biogeography. Taxon.

[6] Neira I., Pobleta L., Porcille P., Silva P., Araya J., Bórquez J., Morales G., Loyola L.A., Sagua H. (1998). Activity of diterpenoids isolated from *Azorella compacta* (Llareta) on *Trypanosoma cruzi* amastigotes. Bol. Chil. Parasitol..

[7] Loyola L.A., Borquez J., Morales G., Araya J., Gonzalez J., Neira I., Sagua H., San-Martín A. (2001). Diterpenoids from *Azorella yareta* and their trichomonicidal activities. Phytochemistry.

[8] Loyola L.A., Borquez J., Morales G., Araya J., Gonzalez J., Neira I., Sagua H., San-Martín A. (2001). Azorellane diterpenoids from Laretia acaulis and its toxoplamacidal activity. Bol. Soc. Chil. Quím..

[9] Loyola L.A., Borquez J., Morales G., San-Martin A., Darias J., Flores N., Gimenez A. (2004). Muliane-type diterpenoids from *Azorella compacta* display antiplasmodial activity. Phytochemistry.

[10] Wächter G.A., Matooq G., Hoffmann J.J., Maiese W.M., Singh M.P., Montenegro G., Timmermann B.N. (1999). Antibacterial diterpenoid acids from *Azorella madreporica*. J. Nat. Prod..

[11] Morales P., Kong M., Pizarro E., Pasten C., Morales G., Borquez J., Loyola L.A. (2003). Effect of azorellanone, a diterpene from Azorella yareta Hauman on human sperm physiology. J. Androl..

[12] Fuentes N.L., Sagua H., Morales G., Borquez J., San-Martín A., Soto J., Loyola L.A. (2005). Experimental antihyperglycemic effect of diterpenoids of llareta *Azorella compacta* (Umbelliferae) Phil in rats. Phytother. Res..

[13] Wächter G.A., Franzblau S.G., Montenegro G., Suarez E., Fortunato R.H., Saavedra E., Timmermann B.N. (1998). A new antitubercular mulinane diterpenoid from *Azorella madreporica* Clos. J. Nat. Prod..

[14] Delporte C., Backhouse N., Salinas P., San-Martin A., Borquez J. (2003). Pharmacotoxicological study of diterpenoids. Bioorg. Med. Chem..

[15] Bórquez J., Loyola L.A., Morales G., San-Martín A., Roldan R., Márquez N., Muñoz E. (2007). Azorellane diterpenoids from *Laretia acaulis* inhibit nuclear factor-kappa B activity. Phytother. Res..

[16] Molina-Salinas G.M., Bórquez J., Ardiles A., Said-Fernández S., Loyola L.A., San-Martín A., González-Collado I., Peña-Rodríguez L.M. (2010). Antituberculosis activity of natural and semisynthetic azorellane and mulinane diterpenoids. Fitoterapia.

[17] Loyola L.A., Bórquez J., Morales G., San-Martín A. (1996). Mulinolic acid, a diterpenoid from *Mulinum crassifolium*. Phytochemistry.

[18] Loyola L.A., Bórquez J., Morales G., San-Martín A. (1997). A new diterpenoid from *Mulinum crassifolium*. Bol. Soc. Chil. Quím..

[19] Frisch M.J., Trucks G.W., Schlegel H.B., Scuseria G.E., Robb M.A., Cheeseman J.R., Scalmani G., Barone V., Mennucci B., Petersson G.A. (2009). Gaussian software.

