# Supplementary material for: Further Mulinane and Azorellane Diterpenoids Isolated from Mulinum crassifolium and Azorella compacta
Source: Molecules. 2014 Mar 28;19(4):3898–908. doi: 10.3390/molecules19043898 (PMC6271538; doi:10.3390/molecules19043898)

Supplementary File

Figure S1. Proton NMR spectra (500.13 MHz) of compound **1.**

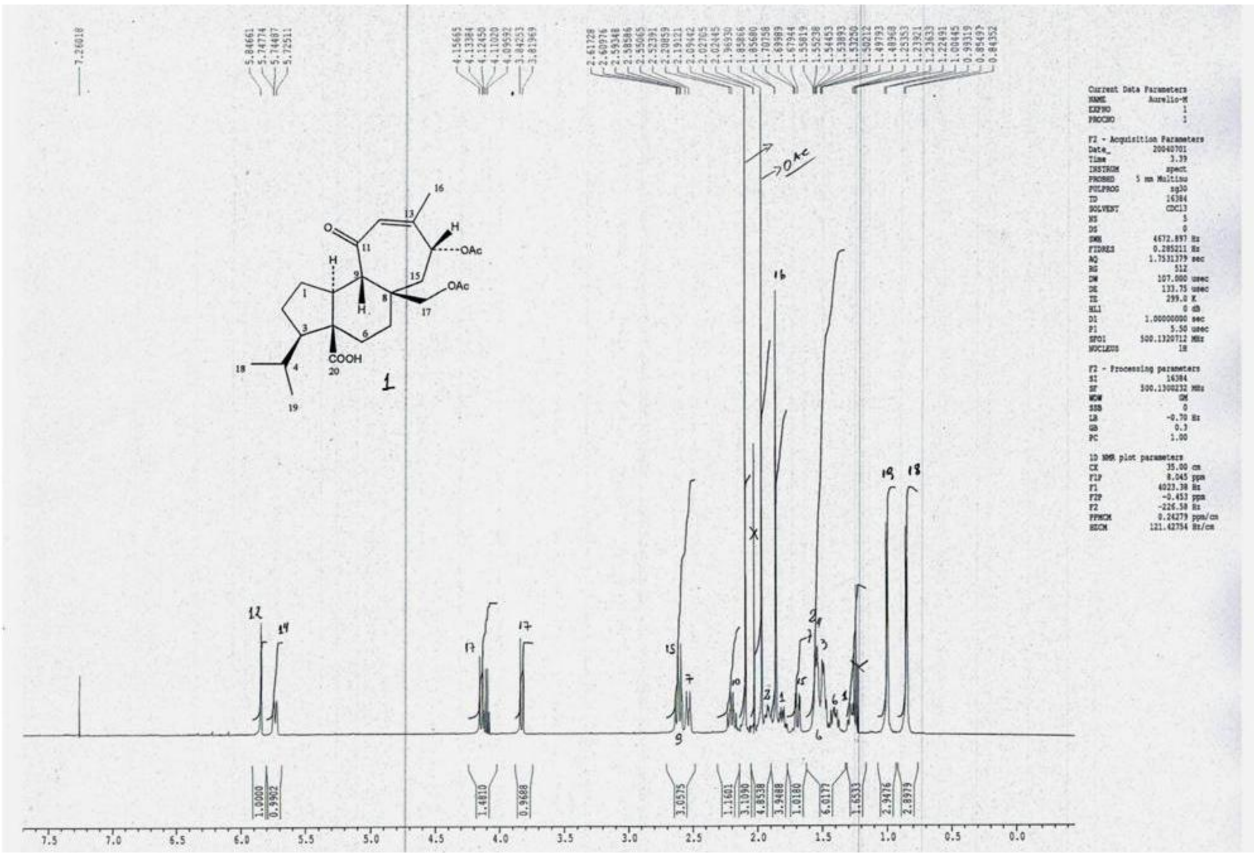

Figure S2. Partial zoom of the proton NMR spectra (500.13 MHz) of compound **1**.

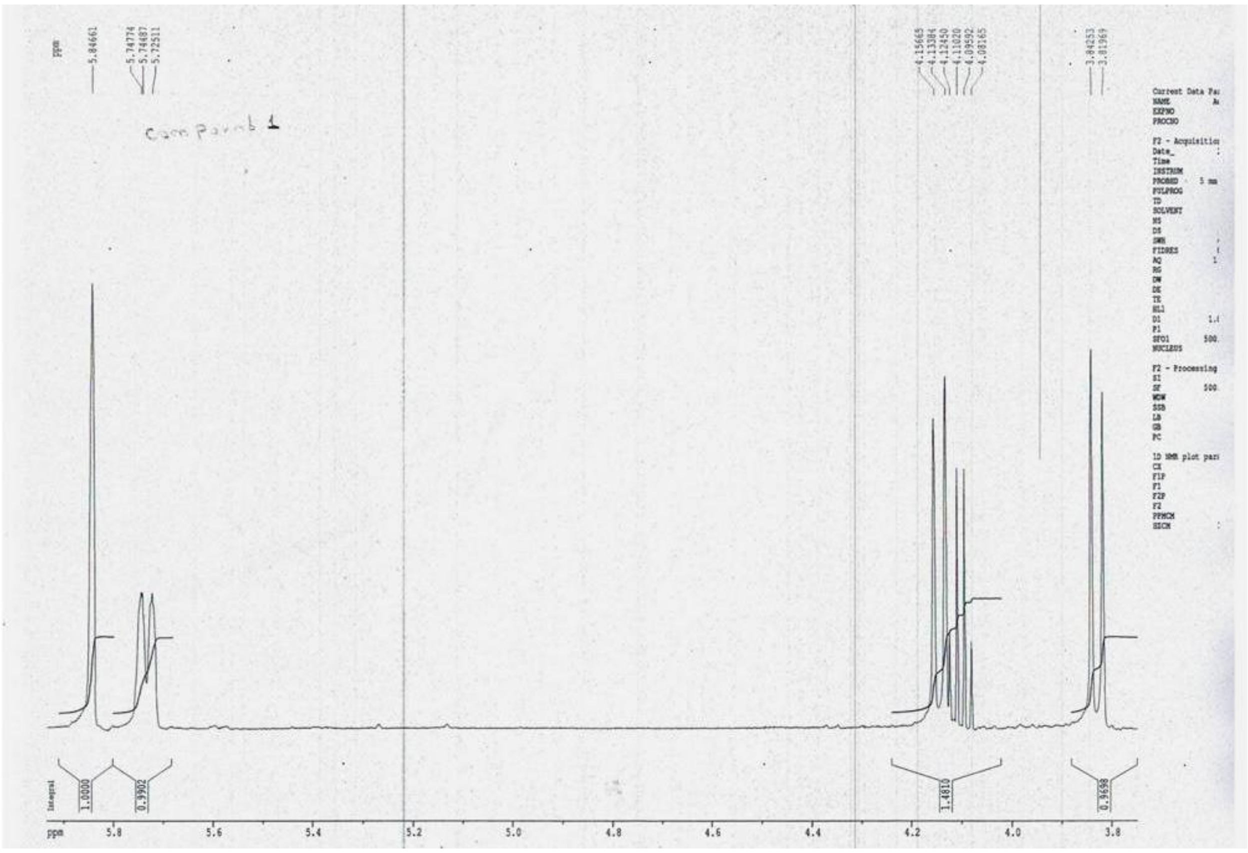

Figure S3. Partial zoom of the proton NMR spectra (500.13 MHz) of compound **1** (Cont.).

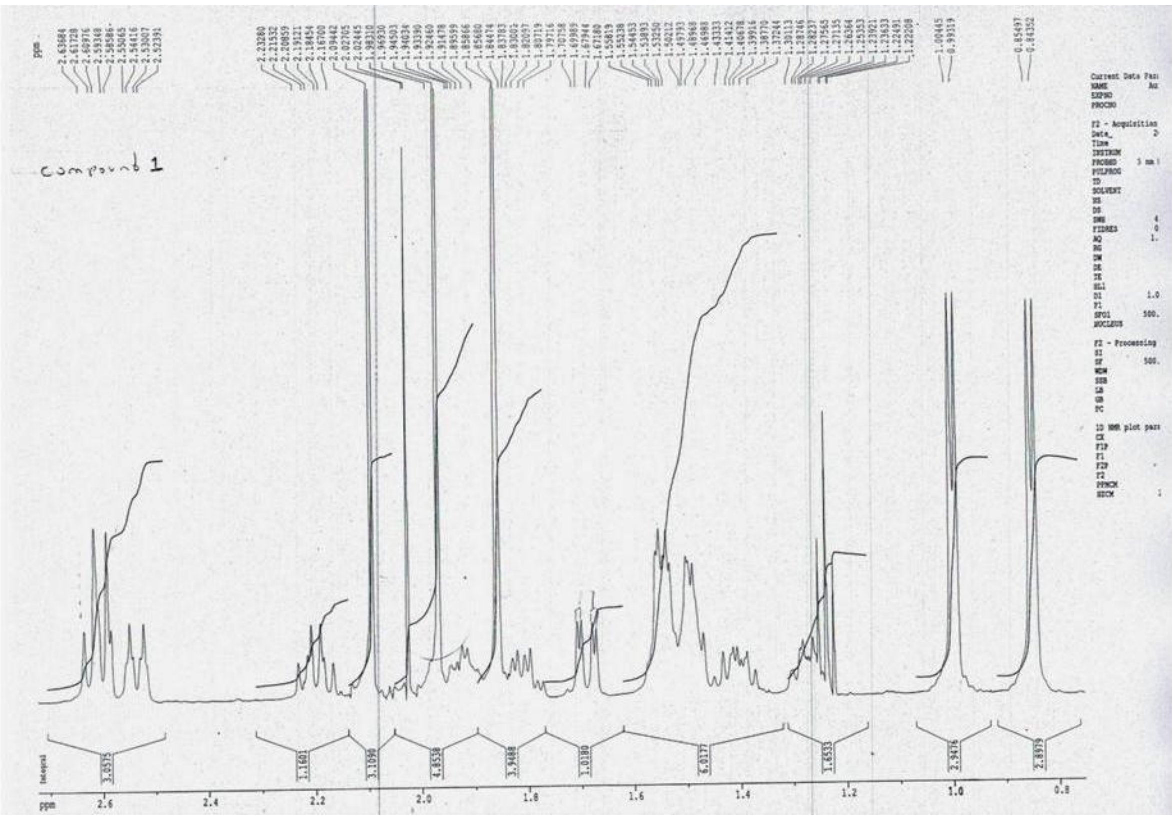

Figure S4. Carbon NMR spectra (125.76 MHz) of compound **1**.

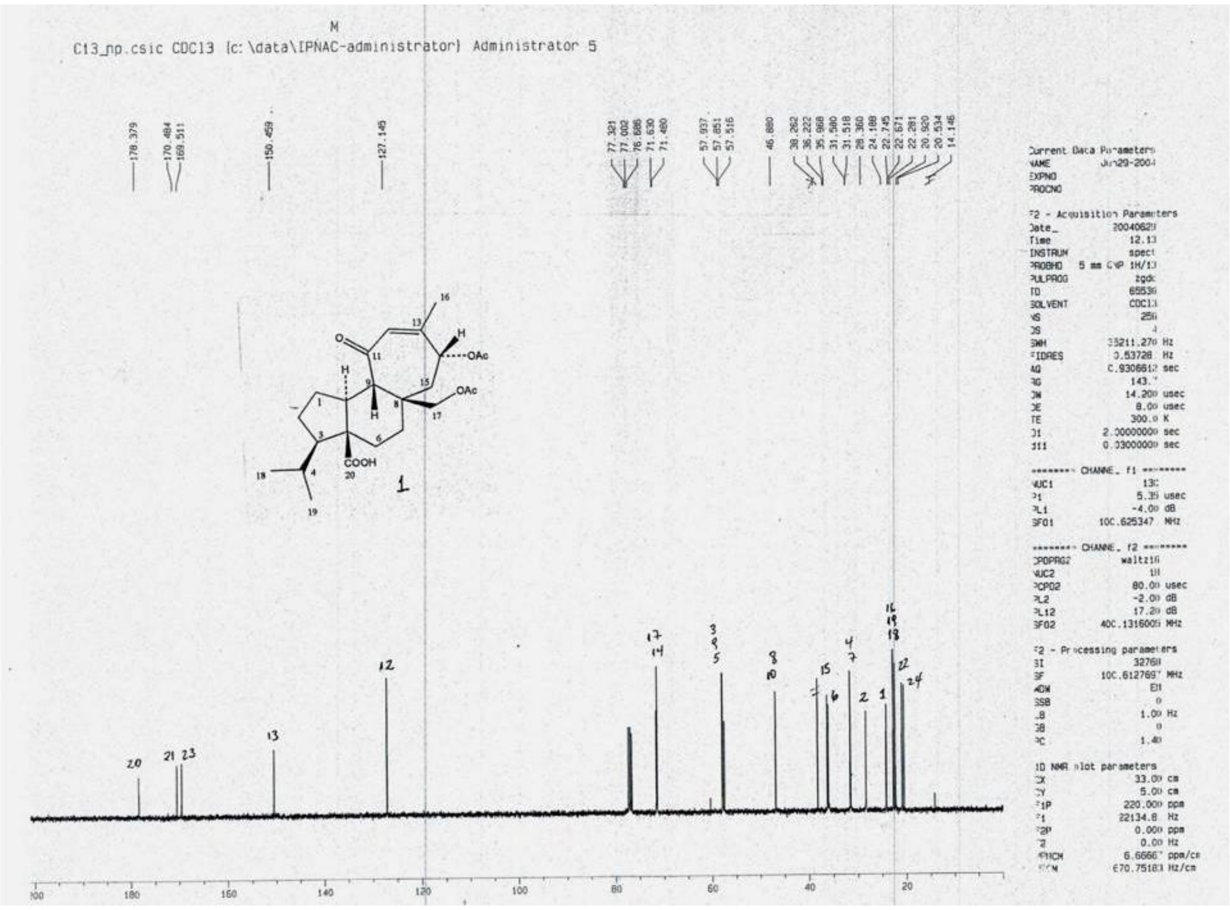

Figure S5. ^{1}H-^{1}H COSY NMR spectra of compound **1**.

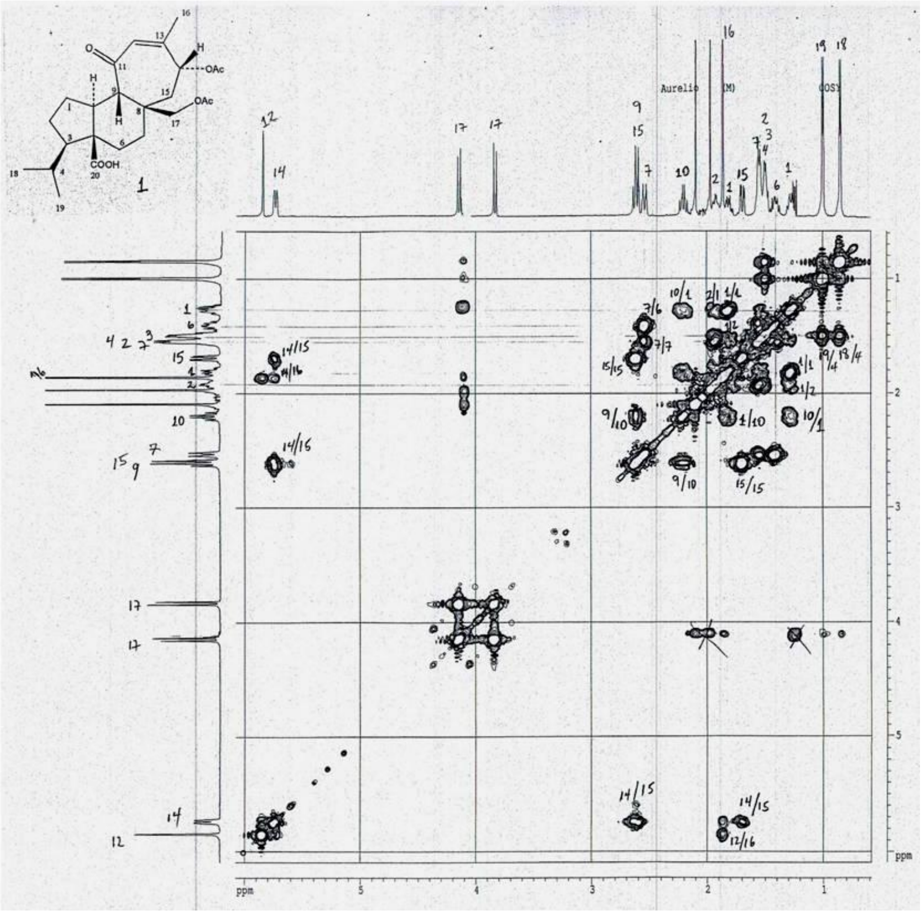

Figure S6. HMBC spectra of compound **1**.

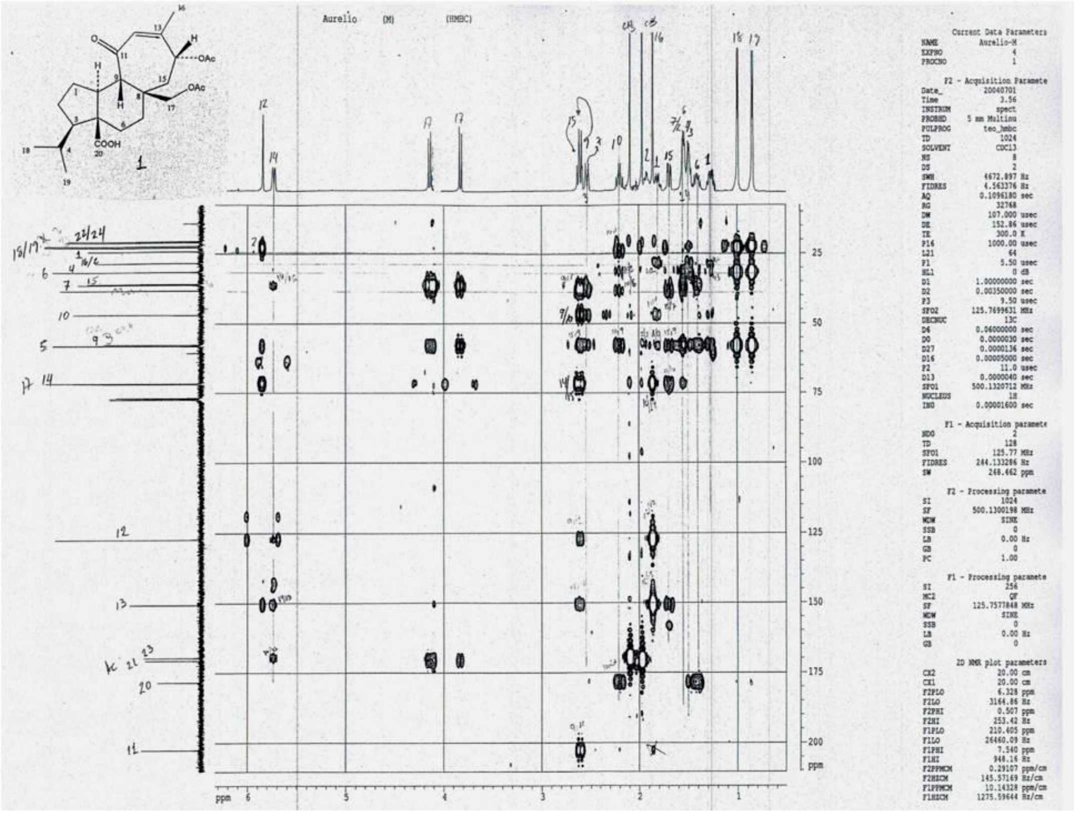

Figure S7. NOESY spectra of compound **1**.

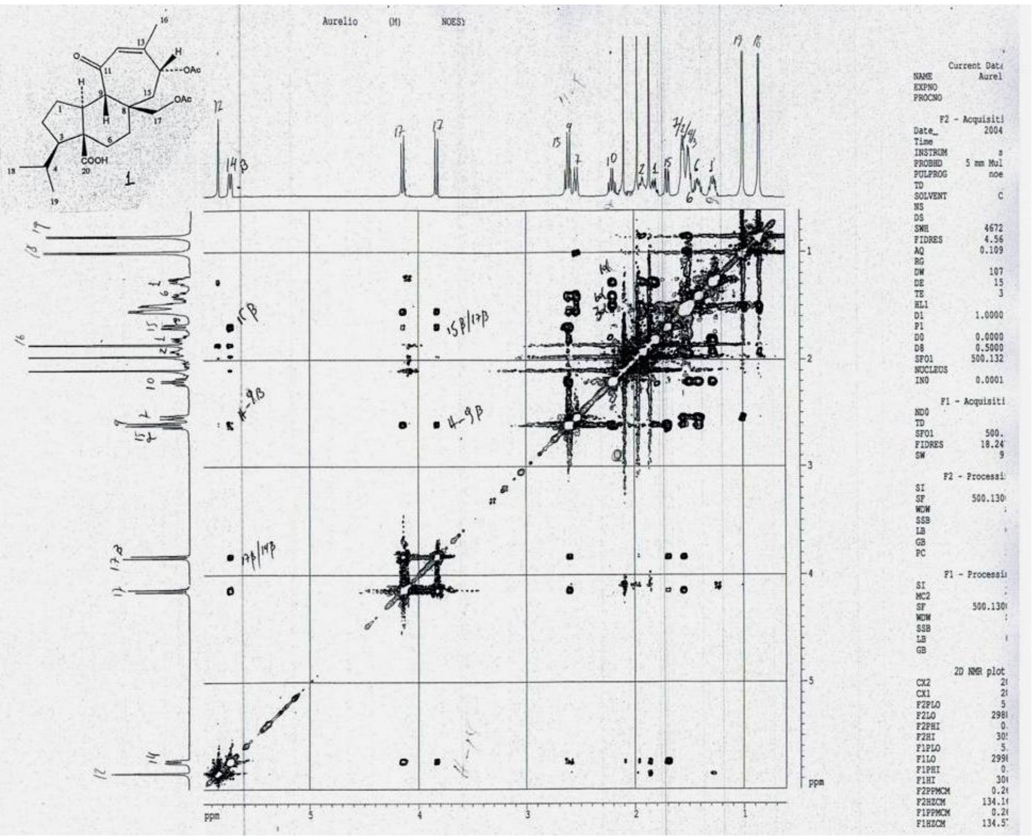

Figure S8. HSQC spectra of compound **1**.

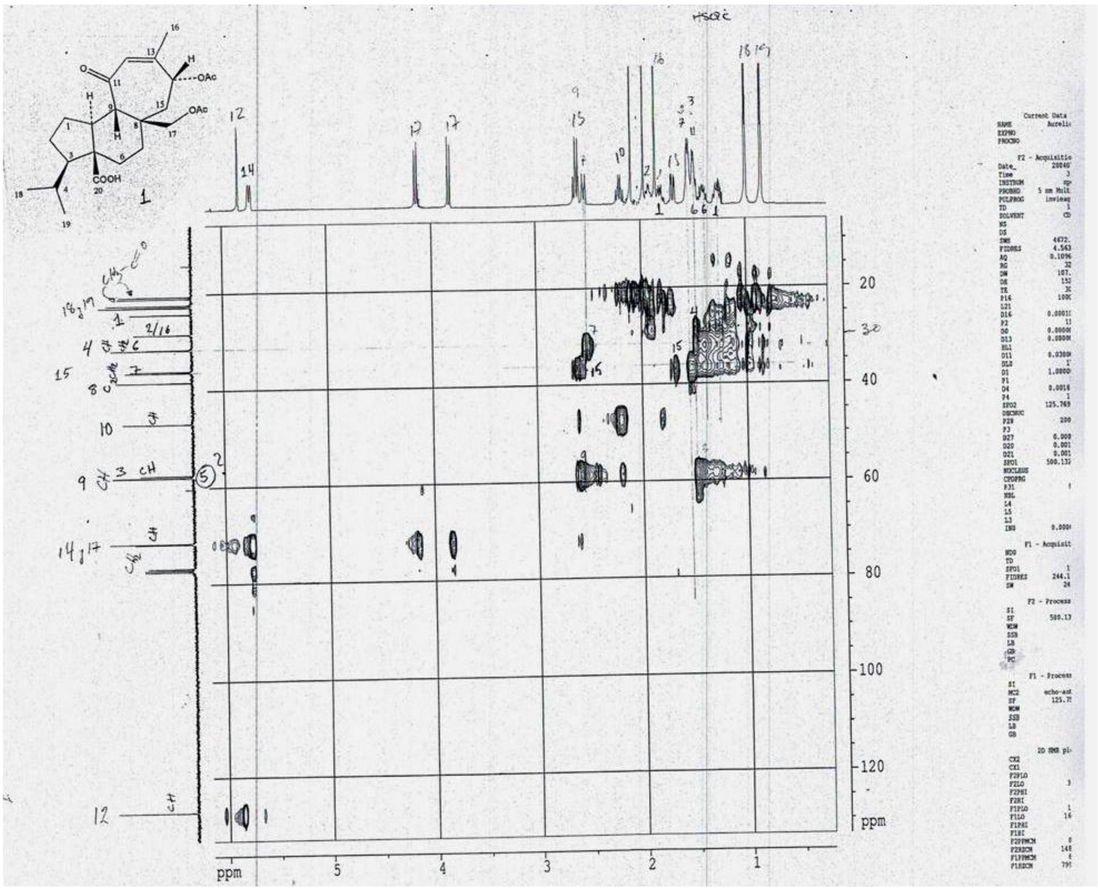

Figure S9. Proton NMR spectra (500.13 MHz) of compound **2**.

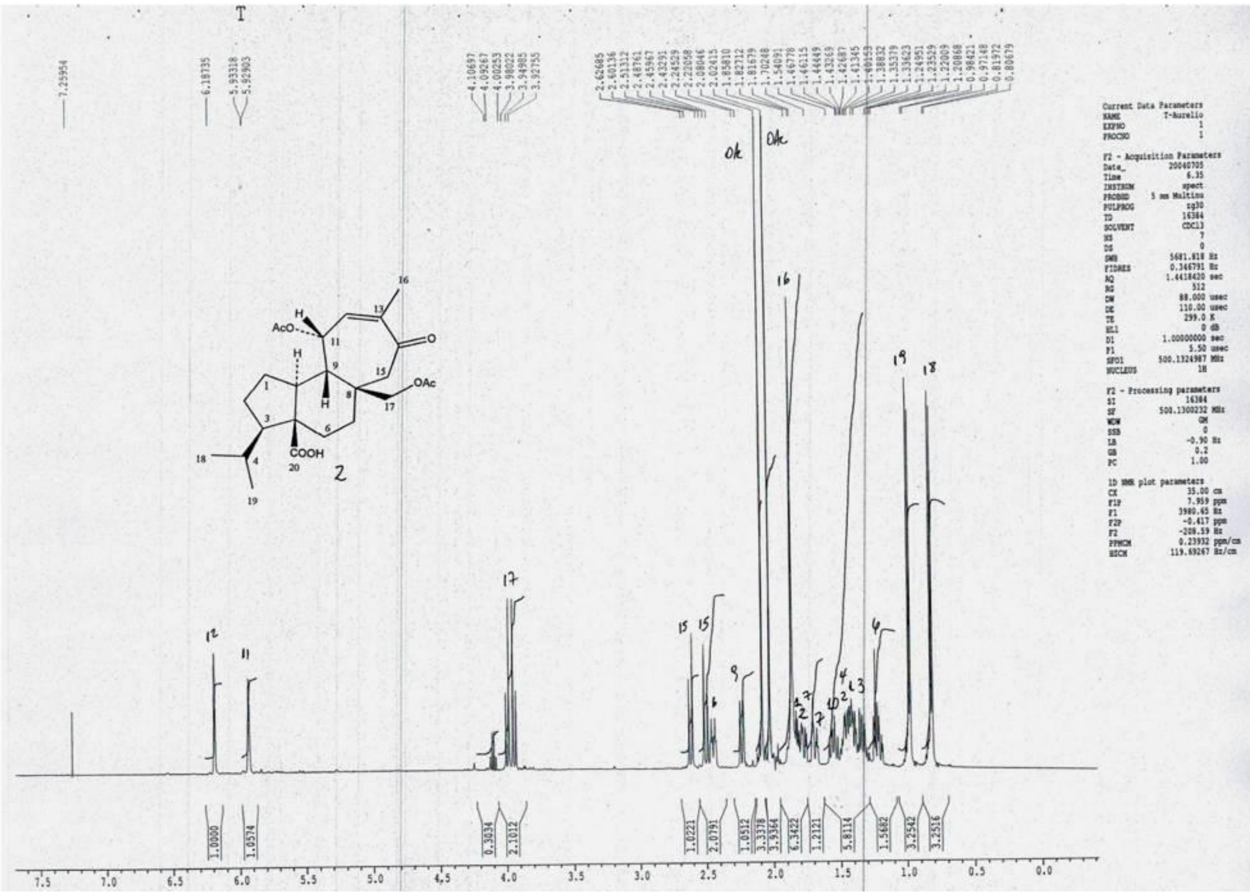

Figure S10. Partial zoom of the proton NMR spectra (500.13 MHz) of compound **2**.

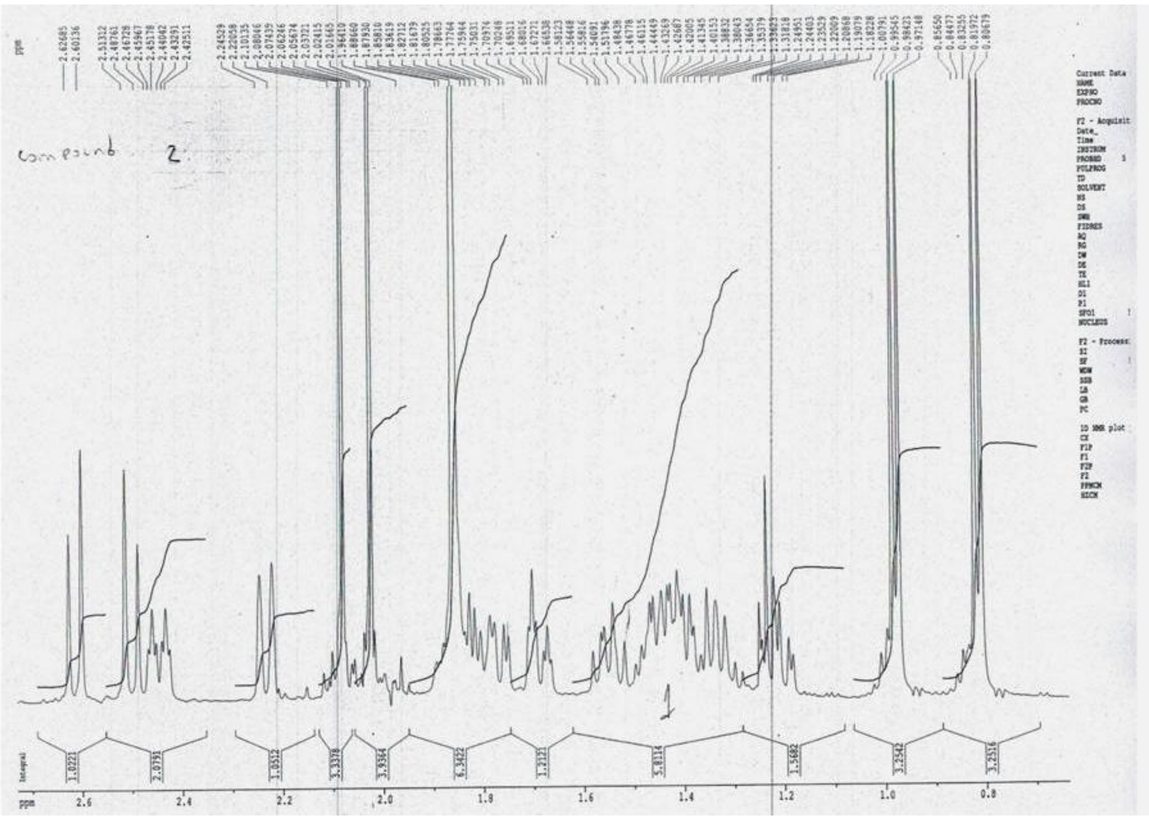

Figure S11. Partial zoom of the proton NMR spectra (500.13 MHz) of compound **2** (Cont).

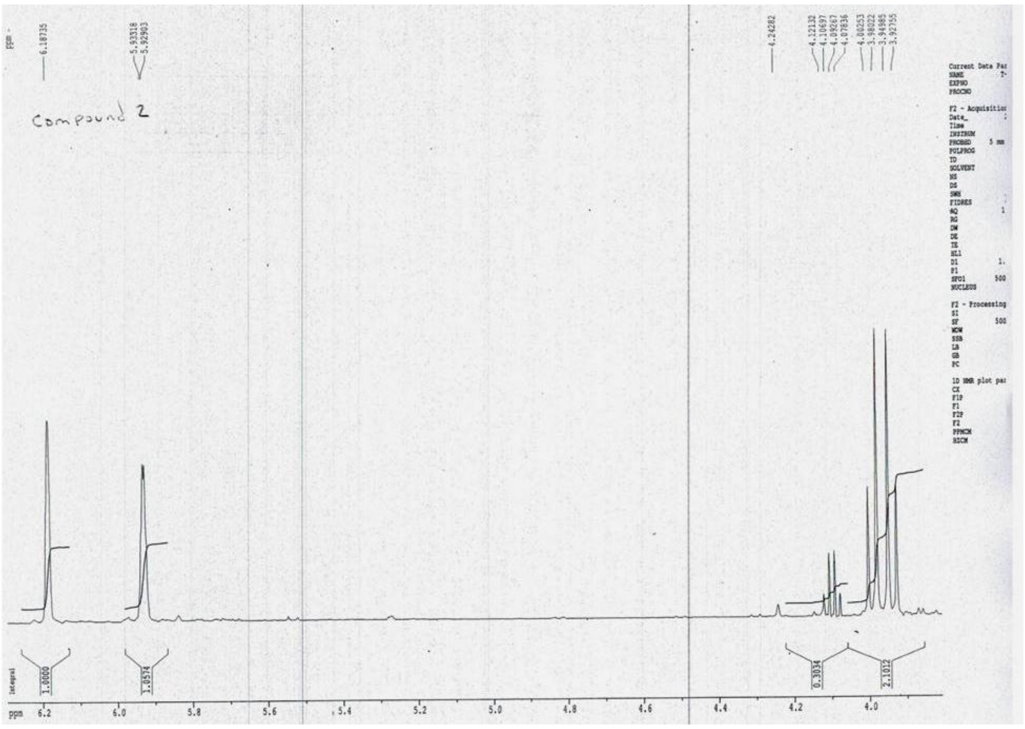

Figure S12. ^{1}H-^{1}H COSY spectra of compound **2**.

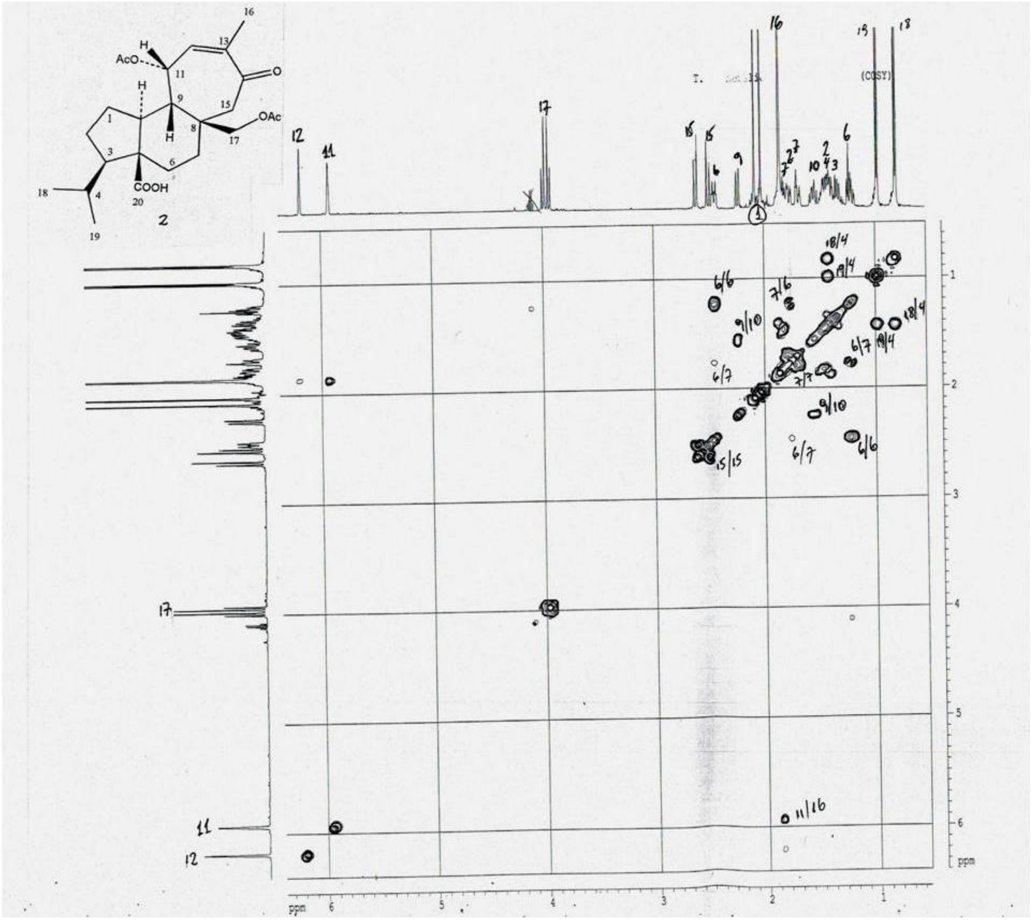

Figure S13. NOESY spectra of compound **2**.

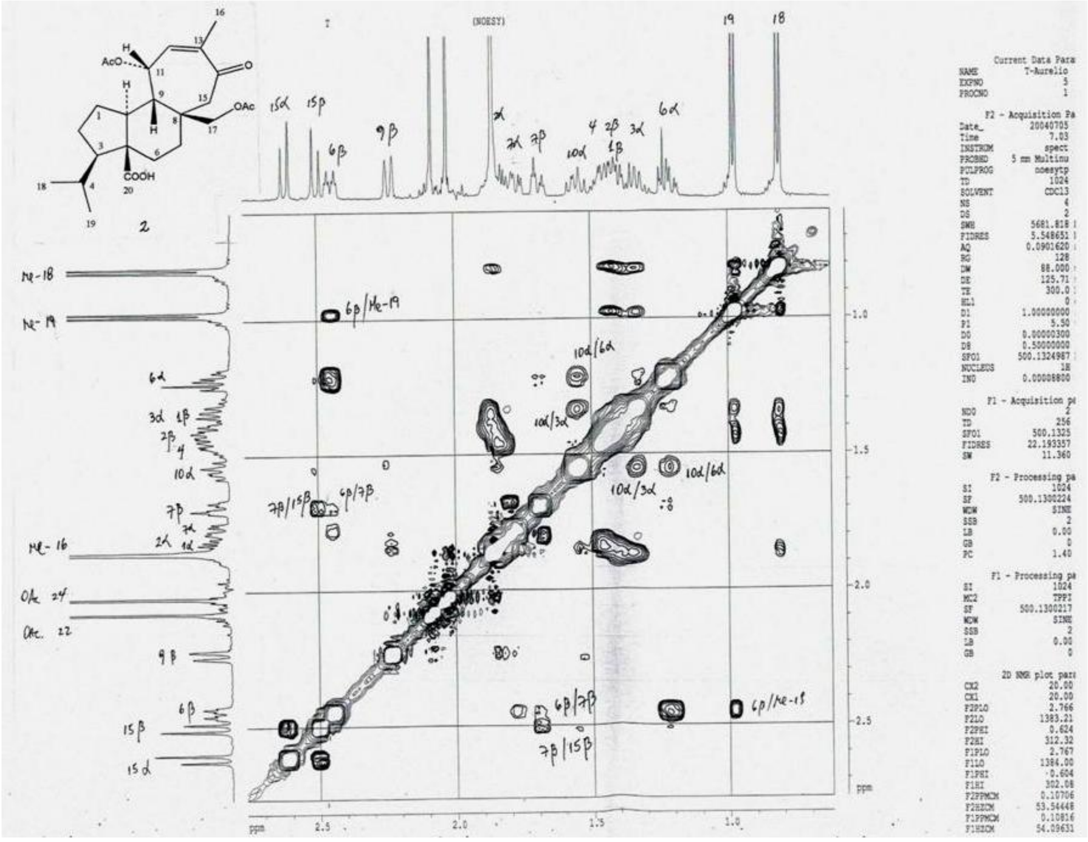

Figure S14. NOESY spectra of compound **2**(Cont.).

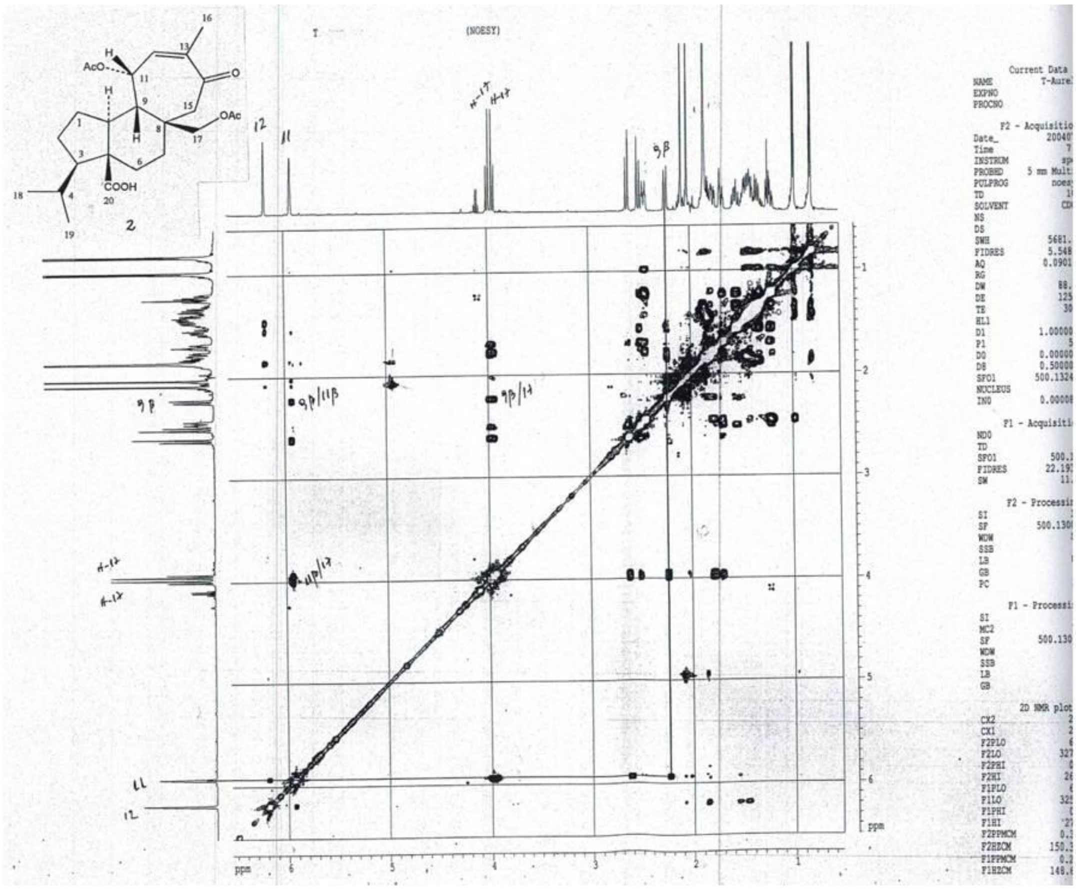

Figure S15. HSQC spectra of compound **2**.

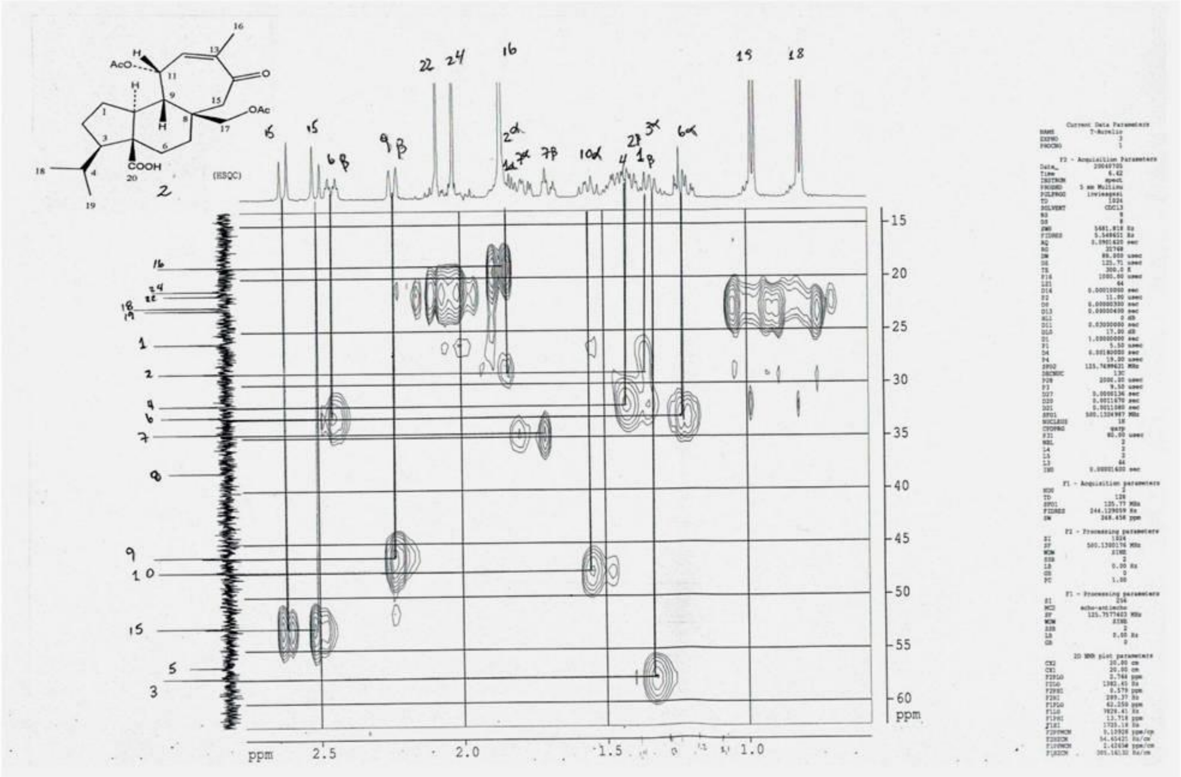

Figure S16. HMBC spectra of compound **2**.

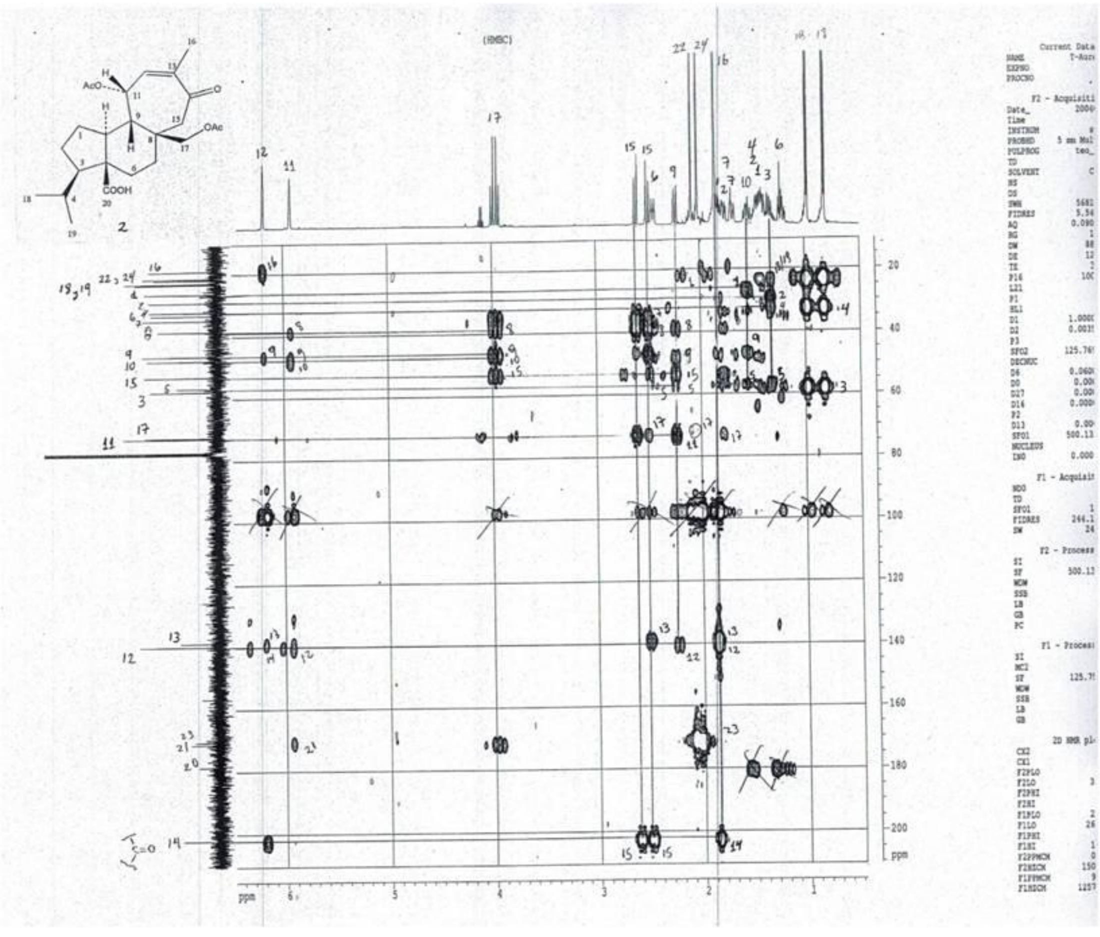

Figure S17. ^{1}H NMR spectra (400.13 MHz) of compound **3**

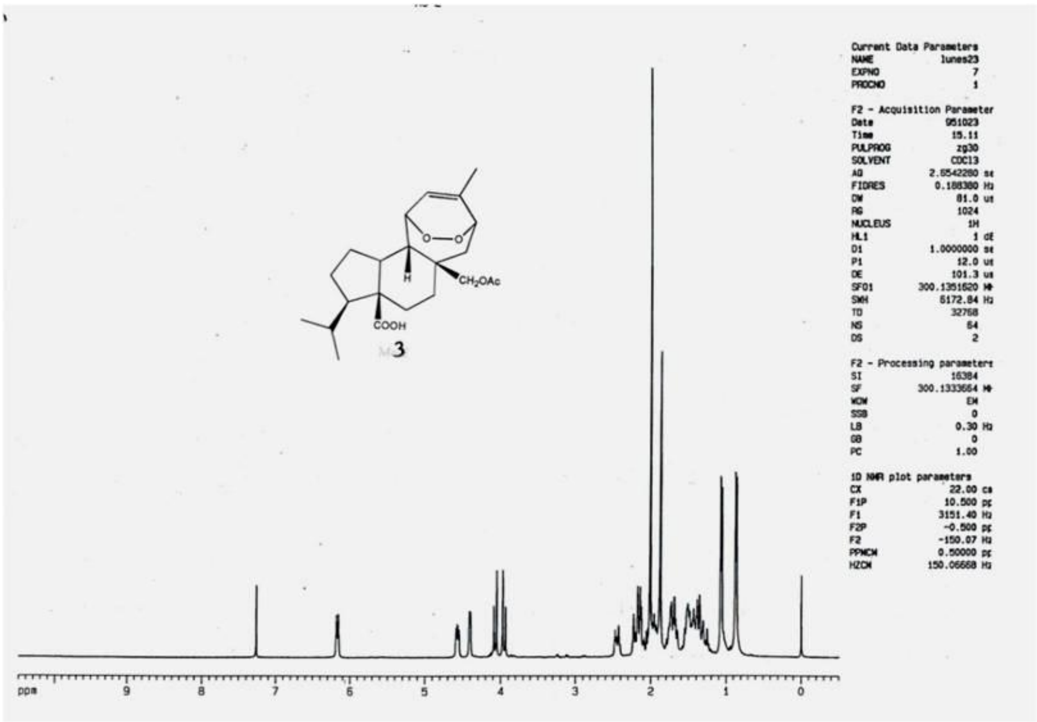

Supplement: Supplementary file 1 [file molecules-19-03898-s001.pdf]
